# Dry sliding wear behavior of SiC-reinforced Al-Si eutectic metal matrix composite

**DOI:** 10.1038/s41598-025-26489-w

**Published:** 2025-11-28

**Authors:** Ashwin Shetty, Thirumaleshwara Bhat, Ananda Hegde, Sathyashankara Sharma, Ravikantha Prabhu

**Affiliations:** 1 Department of Mechanical Engineering, St Joseph Engineering College, Mangaluru, India; 2 Department of Mechanical Engineering, Shri Madhwa Vadiraja Institute of Technology, Bantakal, India; 3 Department of Mechanical and Industrial Engineering, Manipal Institute of Technology, Manipal Academy of Higher Education, Manipal, 576104 Karnataka India

**Keywords:** Magnesium (Mg), Silicon carbide (SiC), Al-Si eutectic alloy, Ultimate tensile brinell wear rate, Coefficient of friction (COF), Taguchi method, ANOVA, Engineering, Materials science

## Abstract

This study investigates the effect of sliding parameters on the dry sliding wear behavior of (silicon carbide) SiC-reinforced (aluminum silicon) Al-Si eutectic metal matrix composites (MMCs) with 1.5 wt% of magnesium (Mg). The composites were fabricated by incorporating varying amounts of SiC particles into the aluminum-silicon matrix to improve wear resistance. A comprehensive wear analysis was conducted under different sliding speeds, normal loads, sliding distances, and SiC reinforcement contents. The Taguchi method was used to optimize the wear performance, with signal-to-noise (SN) ratios employed to assess the influence of each sliding parameter. The findings show that wear rate is mainly influenced by SiC content (32.04%), normal load (30.98%), and sliding speed (10.99%), while the coefficient of friction (COF) is largely affected by normal load (38.51%), SiC content (34.46%), and sliding distance (23.51%). The study also reveals that the addition of SiC reinforcement improves the wear resistance of the developed composite. The findings suggest that optimizing these parameters can significantly reduce wear, making these composites suitable for advanced engineering applications where high wear resistance is critical.

## Introduction

Aluminum–Silicon (Al-Si) eutectic alloys are widely recognized for their excellent castability, lightweight nature, and good mechanical properties, making them suitable for various industrial applications, including the automotive, aerospace, and marine sectors^1–3^. Despite these advantages, their wear resistance under severe service conditions, such as high loads, elevated temperatures, and aggressive sliding, remains limited^4^. To overcome this limitation, reinforcing Al-Si alloys with hard ceramic particles, such as silicon carbide (SiC), Titanium Dioxide (TiO_2_), Boron Carbide (B4C) and coating of alloy has gained significant attention in recent years. Ni–graphene coatings on LM26 aluminum alloy have been shown to improve microhardness, corrosion resistance, and surface durability due to their cauliflower-like morphology, while related advancements such as friction stir spot welding (FSSW) further demonstrate effective strategies for enhancing the structural performance of aluminum alloys^5,6^ . Research on Al7075/TiO_2_/Kaoline hybrid composites has shown that increasing kaoline reinforcement and sintering temperature improves microhardness, lowers friction coefficient, and reduces wear rate^7^. Studies on AA7075/B4C composites report that higher B4C reinforcement improves hardness, strength, and wear resistance, despite some agglomeration at higher levels^8^. Al-Si/Al₂O₃–MoS₂ hybrid composites exhibited enhanced tensile strength, reduced wear, and lower friction. Wear behavior was strongly influenced by contact pressure, and SEM analysis confirmed improved tribological performance, indicating their suitability for demanding automotive applications^9^ Hybrid Al–Si–Mg composites reinforced with MoS₂ and Al₂O₃ were fabricated via stir casting, showing improved hardness, tensile strength, and wear resistance, along with a lower coefficient of friction. SEM analysis indicated adhesive and abrasive wear at low loads and predominantly adhesive wear at high loads, highlighting enhanced tribological performance compared to the base alloy^10^. Using SiC reinforcement significantly improved wear resistance of AlSi alloy^11^,^12^. SiC-reinforced Aluminum matrix composites provide a combination of high strength, stiffness, and superior wear properties, making them highly desirable for applications requiring enhanced tribological performance^12,13^. The inclusion of SiC in Al-Si alloys enhances mechanical properties and wear resistance by increasing hardness, reducing friction, and minimizing plastic deformation under load ^14–16^. The hard ceramic particles act as a barrier to material removal during wear, thereby limiting surface damage ^16^.

The development of aluminum matrix composites reinforced with ceramic particles such as SiC has gained significant attention in recent years, particularly for applications requiring enhanced mechanical strength and wear resistance. A critical factor influencing the performance of these composites is the addition of alloying elements like magnesium (Mg), which has been shown to considerably improve the interfacial bonding between the aluminum matrix and the reinforcement phase. Mg enhances the wettability of SiC particles, promoting uniform dispersion and stronger matrix-reinforcement adhesion. Furthermore, Mg contributes to the formation of a stable surface oxide layer, which plays a crucial role in resisting material degradation under sliding conditions^17,18^. During the solidification process in casting, the dissolution of Mg encourages the formation of Mg₂Si precipitates, which are known to significantly enhance hardness and improve wear resistance. However, these beneficial effects are highly dependent on the controlled addition of Mg, as excessive concentrations may alter the phase balance and negatively impact the composite’s structural properties. Prior studies have emphasized the importance of optimizing Mg content to maximize mechanical performance and tribological stability ^17^. Microstructural investigations, supported by optical and scanning electron microscopy, have shown that higher Mg content favors the formation of strengthening Mg₂Si phases while suppressing the presence of brittle intermetallic compounds such as Al₅FeSi and Al₈Si₆Mg₃Fe, which are often detrimental to overall composite performance^19–21^. Study on wear behavior of SiC-reinforced Al-Si eutectic composites containing traces of magnesium, revealing that the incorporation of ceramic particles significantly enhanced strength, thermal stability, and interfacial bonding, while the formation of an Mg₂C interphase improved resistance to oxidation and corrosion^22^. In addition to compositional factors, external tribological parameters, such as sliding speed, applied load, sliding distance, and the proportion of reinforcement, also play a decisive role in determining wear characteristics. It has been widely reported that increases in sliding speed and load typically lead to higher wear rates due to the generation of frictional heat and plastic deformation of the matrix material^23,24^. Nevertheless, the presence of SiC particles mitigates these adverse effects by providing surface reinforcement, thereby improving resistance to wear under demanding conditions^25–27^. Together, these findings highlight the critical need to optimize both compositional and operational parameters when designing aluminum-based composites for high-performance, wear-resistant applications.

The Taguchi Design of Experiments (DOE) is an established statistical methodology that facilitates the systematic examination of the influence of multiple input factors on the performance of a system or process^28,29^. This approach is characterized by the use of orthogonal arrays, which enable researchers to design efficient and balanced experiments with a reduced number of trials while still obtaining reliable and comprehensive data^28,30^. A key feature of the Taguchi method is the application of signal-to-noise (S/N) ratio analysis, which, when coupled with analysis of variance (ANOVA), allows for the quantification of the contribution of individual control factors to the variability in the response variable^31–33^. This analytical framework supports the identification of robust process parameters that can yield consistent performance despite the presence of external noise or uncertainty. The Taguchi method is particularly valued for its ability to optimize experimental conditions with minimal resource expenditure, thereby accelerating the pace of research and development activities. In the field of materials engineering, particularly in wear analysis, the method has demonstrated considerable effectiveness^28,30^. It has been successfully applied to investigate the dry and abrasive wear behavior of polymer matrix composites, where it enabled the evaluation and ranking of factors influencing wear resistance under various testconditions^34,35,33,29^. Similarly, in the study of MMCs, the Taguchi approach has been instrumental in analyzing the complex interactions between process parameters and material performance, facilitating the development of optimized composite formulations with improved wear characteristics^31,36,37^.These applications underscore the method’s versatility and reliability in materials research, making it a preferred tool for experimental optimization in both academic and industrial settings.

Although significant research has been conducted on the wear behavior of SiC-reinforced Al–Si metal matrix composites, there remains a lack of detailed understanding regarding the influence of specific sliding parameters on wear performance under dry sliding conditions. Investigating the relationship between factors such as sliding speed, normal load, and reinforcement content is essential for improving the wear resistance of these composites. The incorporation of SiC particles, along with trace amounts of Mg, provides a promising approach to enhancing hardness and tribological properties in high-wear environments. This study addresses these gaps by systematically examining the effects of sliding speed, normal load, and SiC content on the dry sliding wear behavior of Al–Si–1.5 Mg eutectic MMCs. Additionally, a Taguchi design of experiment has been employed to optimize the process parameters and identify the most influential factors affecting wear and friction behavior, highlighting the novelty and structured approach of the research.

## Materials and experimental details

### Material selection

The study examines a eutectic Al-Si alloy that contains approximately 12 wt% silicon. The matrix material’s chemical composition includes 11.72 wt% silicon (Si), 0.02 wt% zinc (Zn), 0.01 wt% manganese (Mn), 0.03 wt% copper (Cu), and 0.06 wt% iron (Fe), with aluminum (Al) constituting the remaining balance. Commercially sourced magnesium (Mg) powders, with a particle size of 50 μm, were used for dissolution in the eutectic alloy, with 1.5 wt% Mg added to the aluminum alloy. For the preparation of the composite, SiC particles, ranging in size from 60 to 80 μm, served as the reinforcing material^9^. The SiC particle are added in the wt% of 0, 2 and 4.

### Composite preparation

The composite specimens were fabricated using the conventional stir-casting technique, a well-established method for producing particle-reinforced metal matrix composites due to its simplicity and cost-effectiveness ^39–41^. The process was conducted in two stages to ensure homogeneous distribution of the reinforcements. In the first stage, aluminum ingots were sectioned and placed into a graphite crucible, which was then introduced into an electric resistance furnace maintained at 700 °C. Once fully molten, hexachloroethane powder was added to the melt to remove dissolved gases and impurities.

In the second stage, the furnace temperature was reduced to 600 °C, and magnesium (1.5 wt.%) wrapped in aluminum foil was introduced to the melt to enhance wettability and interfacial bonding between the matrix and the reinforcement. Mechanical stirring was carried out at 600 RPM using a stainless-steel impeller. Simultaneously, SiC particles preheated to 200 °C were gradually added to the slurry in varying weight fractions (0, 2, and 4 wt.%) to prevent thermal shock and to promote better incorporation. The stirring continued for 5–7 min to ensure uniform dispersion of the reinforcement throughout the melt^42–44^. The resulting mixture was poured into preheated steel molds and allowed to solidify under ambient conditions. The cast samples were then machined to the required dimensions in accordance with ASTM standards for subsequent characterization and testing.

**Fig. 1:**
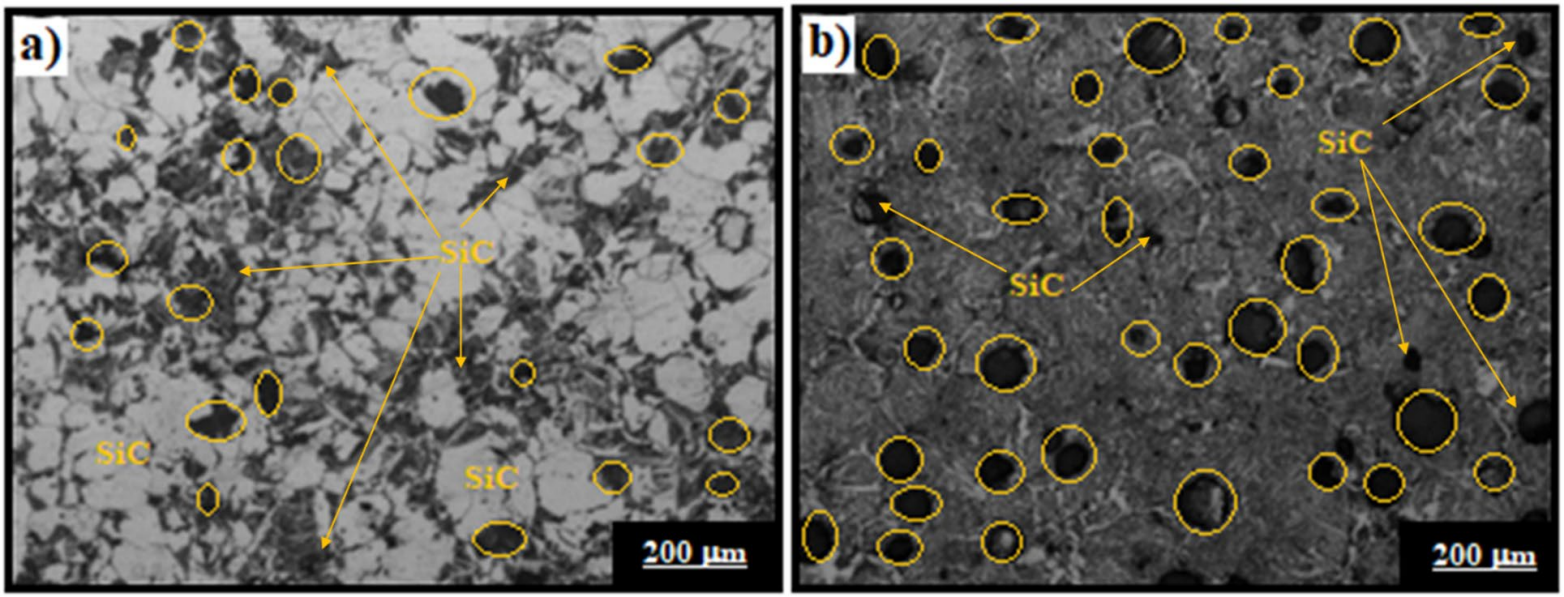
Optical microscopic images of (**a**) AlSi-1.5 Mg-2SiC (**b**) AlSi-1.5 Mg-4SiC as cast samples.

Microstructural images of the as-cast composites was taken using an OLYMPUS-U-TV0.5XC-3 optical microscope. As seen in Fig. 1, the contrast difference comes from the amount of SiC present in the composites. In the AlSi-1.5 Mg-2SiC sample (Fig. 1a), the lower particle content makes the SiC less noticeable within the matrix. On the other hand, the AlSi-1.5 Mg-4SiC sample (Fig. 1b) shows a higher concentration of SiC, which appears darker and stands out more clearly. This sharper contrast at higher reinforcement levels also indicates that the particles are well distributed and strongly bonded to the matrix, with no visible casting defects.

### Characterization of composite

**Fig. 2:**
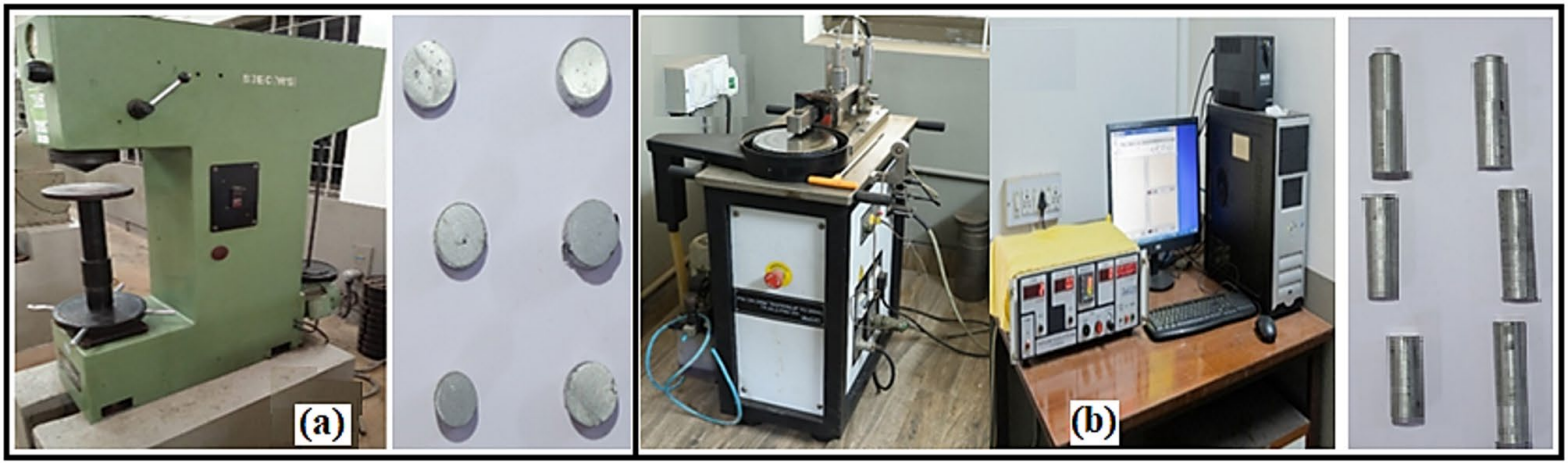
Digital image of (**a**) Brinell hardness tester with specimen (**b**) Pin-on-Disc wear tester with sample specimen.

To evaluate the mechanical and tribological behavior of the fabricated composite specimens, a series of standardized tests were carried out. The density was measured as per ASTM B311 standard using Archimedes’ principle. The hardness of the samples was measured using a Brinell hardness tester in accordance with the ASTM E10-00 standard. The test employed a 5 mm diameter steel ball indenter under an applied load of 250 kgf, ensuring reliable and consistent results across all specimens. For assessing the sliding wear characteristics, experiments were performed using a Pin-on-Disk type wear and friction monitoring system equipped with a digital data acquisition interface, conforming to ASTM G99 guidelines. Prior to testing, all samples were thoroughly cleaned using an ethanol solution to eliminate surface contaminants that could affect weight measurement accuracy. The test specimens were cylindrical in shape, with a diameter of 8 mm and a height of 30 mm, and were mounted securely on the pin holder during testing. The experimental setup used for hardness and wear testing is shown in Fig. 2, which presents (a) the Brinell hardness tester with specimen and (b) the Pin-on-Disc wear tester with sample specimen.

The wear tests involved systematic variation of key tribological parameters, including sliding speed, normal load, and sliding distance, while maintaining a constant track diameter of 60 mm. Each specimen was subjected to a preliminary run-in period of 10 min to promote proper contact alignment between the sliding surfaces, thereby minimizing the influence of initial surface roughness. Throughout the tests, the weight loss due to wear was recorded to compute the wear rate, and the coefficient of friction (COF) was continuously monitored using the integrated data acquisition system. The selected levels of wear test parameters and their combinations used in the experiments are summarized in Table 1. This comprehensive approach ensured a robust evaluation of the composite’s wear resistance under varying operational conditions.

### Statistical analysis

Statistical analysis is conducted using the MINITAB 17 statistical software package. The impact of 4 control variables, each at 3 levels, is explored using a Taguchi L_27_(3^45^) orthogonal array design^46,47^.

The S/N ratio functions as a statistical metric within the framework of the Taguchi method. Derived from empirical observations, the S/N ratio corresponds to the "lower the better" (LB) attribute when analyzing wear rate and COF. ANOVA was conducted to determine the proportionate influence of individual control variables and their interactive effects on the wear rate and COF. Additionally, a predictive model based on Taguchi’s approach was formulated to confirm the relationship between test parameters and performance output ^48^.

**Table 1:** Control variable and their levels.

Factor	Type	Levels	Values
Sliding speed (RPM)	Fixed	3	150, 300, 450
Normal load (N)	Fixed	3	15, 30, 45
SiC (wt %)	Fixed	3	0, 2, 4
Sliding distance (m)	Fixed	3	1500, 3000, 4500

## Findings and discussion

### Density and hardness

The Table 2 presents the density, void fraction and Brinell Hardness Number (BHN) of aluminum-silicon (AlSi) alloys with 1.5 wt% Mg, reinforced with different wt% of SiC particles.

**Table 2:** Density, void fraction and hardness.

Material	Density(g/cc)		Void fraction(%)	BHN
	Measured	Theoretical		
AlSi-1.5Mg-0SiC	2.6583	2.571	3.2840	127
AlSi-1.5Mg-2SiC	2.66704	2.6	2.5136	139
AlSi-1.5Mg-4SiC	2.67583	2.571	3.9176	157

The density, void fraction, and hardness of the composite materials reveal significant insights into their structural and mechanical properties. The measured densities of the composites show a gradual increase with the addition of SiC particles: 2.6583 g/cc for the AlSi-1.5 Mg-0SiC composite, 2.66704 g/cc for the AlSi-1.5 Mg-2SiC composite, and 2.67583 g/cc for the AlSi-1.5 Mg-4SiC composite. This trend can be attributed to the higher density of SiC (3.1 g/cc) compared to aluminum, which contributes to an overall increase in density as more SiC is incorporated into the matrix. The void fraction percentages remain relatively low, with 3.2840% for the AlSi-1.5 Mg-0SiC composite, decreasing to 2.5136% for the AlSi-1.5 Mg-2SiC composite, and slightly increasing to 3.9176% for the AlSi-1.5 Mg-4SiC composite. The variations in void fraction can be linked to the processing conditions during composite preparation, such as the mixing and sintering stages, which may affect the consolidation of the material and the presence of voids. The BHN shows a notable increase with SiC content, rising from 127 for the AlSi-1.5 Mg-0SiC composite to 157 for the AlSi-1.5 Mg-4SiC composite. This enhancement in hardness is likely due to the reinforcing effect of SiC particles, which improve the material’s resistance to deformation. Similar observations have been made in earlier studies, where the incorporation of SiC into aluminum matrix composites was found to significantly improve mechanical properties^49^. The addition of ceramic particles has also been reported in previous studies to enhance the mechanical and wear properties of alloys^50^,^51^. The enhancement in hardness, along with noticeable changes in density and porosity, was attributed to the combined effect of material characteristics and the processing techniques used during fabrication^52–54^.

### Statistical analysis

The Taguchi DOE approach was employed to evaluate the experimental results of the AlSi eutectic alloy composite reinforced with SiC. The Table 3 shows the results of the Taguchi L27 experimental design, where each trial represents a specific combination of sliding speed, normal load, SiC content, and sliding distance. For every trial, the mean wear rate and COF were calculated from repeated measurements to ensure accuracy, and the SN ratios are reported for performance evaluation. Standard deviation and repeatability were monitored to confirm the consistency and reliability of the measurements. This method allows for systematic optimization of process parameters and helps identify the factors that have the greatest influence on wear behavior. For each parameter set, four trials were conducted, and the mean values were considered for analysis. The average S/N ratios for wear rate and COF were found to be 40.93 dB and 8.18 dB, respectively.

**Table 3:** Test conditions and output results.

Trial No.	Sliding speed (RPM)	Normal load (*N*)	SiC (wt%)	Sliding Distance (m)	Mean Wear rate (mm^3^/Nm)	SN Ratio	COF	SN Ratio
1	150	15	0	1500	0.008002	41.9360	0.3865	8.25701
2	150	15	2	3000	0.007959	41.9828	0.4053	7.84494
3	150	15	4	4500	0.005499	45.1943	0.4264	7.40343
4	150	30	0	3000	0.009581	40.3718	0.3812	8.37769
5	150	30	2	4500	0.008554	41.3566	0.4000	7.95976
6	150	30	4	1500	0.007654	42.3222	0.3952	8.06439
7	150	45	0	4500	0.009687	40.2762	0.3815	8.37036
8	150	45	2	1500	0.009957	40.0374	0.3744	8.53430
9	150	45	4	3000	0.007887	42.0618	0.3935	8.10159
10	300	15	0	3000	0.010027	39.9766	0.3918	8.13944
11	300	15	2	4500	0.008417	41.4969	0.4106	7.73255
12	300	15	4	1500	0.005997	44.4413	0.4038	7.87738
13	300	30	0	4500	0.011612	38.7019	0.3864	8.25827
14	300	30	2	1500	0.007359	42.6636	0.3793	8.42009
15	300	30	4	3000	0.009289	40.6406	0.3984	7.99289
16	300	45	0	1500	0.010681	39.4278	0.3578	8.92613
17	300	45	2	3000	0.010514	39.5646	0.3796	8.41297
18	300	45	4	4500	0.010144	39.8758	0.3988	7.98586
19	450	15	0	4500	0.010771	39.3549	0.3981	8.00112
20	450	15	2	1500	0.008094	41.8367	0.3909	8.15820
21	450	15	4	3000	0.005984	44.4602	0.4100	7.74339
22	450	30	0	1500	0.011937	38.4621	0.3638	8.78248
23	450	30	2	3000	0.009407	40.5310	0.3856	8.27751
24	450	30	4	4500	0.010517	39.5622	0.4047	7.85686
25	450	45	0	3000	0.011272	38.9600	0.3671	8.70379
26	450	45	2	4500	0.011699	38.6370	0.3859	8.27026
27	450	45	4	1500	0.008849	41.0621	0.3791	8.42442

The analysis of wear rate and COF responses with respect to SiC particle-filled AlSi composite is detailed in Table 4. The response table for SN ratios, when applied to the wear rate, shows that SiC content has the most significant impact, with the highest delta value of 2.46. This indicates that increasing the SiC content substantially reduces the wear rate, likely due to its role in enhancing the material’s resistance to wear^27^. Normal load ranks second with a delta of 2.31, suggesting that both very low and very high loads can effectively reduce the wear rate, as indicated by the higher SN ratios at these levels. Sliding speed has a moderate effect, with a delta of 1.41, and the wear rate decreases at lower sliding speeds, where the highest SN ratio (41.73) is observed, implying that slower speeds help minimize wear. Sliding distance has the least effect on wear rate, with a delta of 0.86, where shorter distances show a slightly higher SN ratio (41.35), indicating less wear over shorter sliding distances. In summary, increasing SiC content, operating at optimal loads, and reducing both sliding speed and distance contribute to a lower wear rate.

**Table 4:** Response table of wear rate and COF.

Level	Wear rate				COF			
	Sliding speed (RPM)	Normal load (*N*)	SiC (wt%)	Sliding distance (m)	Sliding speed (RPM)	Normal load (*N*)	SiC (wt%)	Sliding distance (m)
1	41.73	42.30	39.72	41.35	8.101	7.906	8.424	8.383
2	40.75	40.51	40.90	40.95	8.194	8.221	8.179	8.177
3	40.32	42.18	39.99	40.50	8.246	8.414	7.939	7.982
Delta	1.41	2.31	2.46	0.86	0.145	0.508	0.485	0.401
Rank	3	2	1	4	4	1	2	3

Table 4 reveals that normal load has the most significant impact on COF, with the highest delta value (0.508), indicating it plays a crucial role in reducing COF. The lowest COF is observed at level 3 (8.414) of normal load, meaning that lower loads contribute to better friction performance. SiC content ranks second with a delta of 0.485, showing that increasing SiC content to level 3 (7.939) leads to a notable increase in COF. Sliding distance ranks third, with a delta of 0.401, where shorter distances correspond to a lower COF, as shown by the SN ratio at level 3 (7.982). Sliding speed has the least impact, with a delta of 0.145, although higher speeds result in slightly better frictional behavior, as seen at level 3 (8.246). Overall, reducing normal load and sliding distance, while increasing SiC content, effectively lowers COF and improves friction performance^55,56^.

**Fig. 3:**
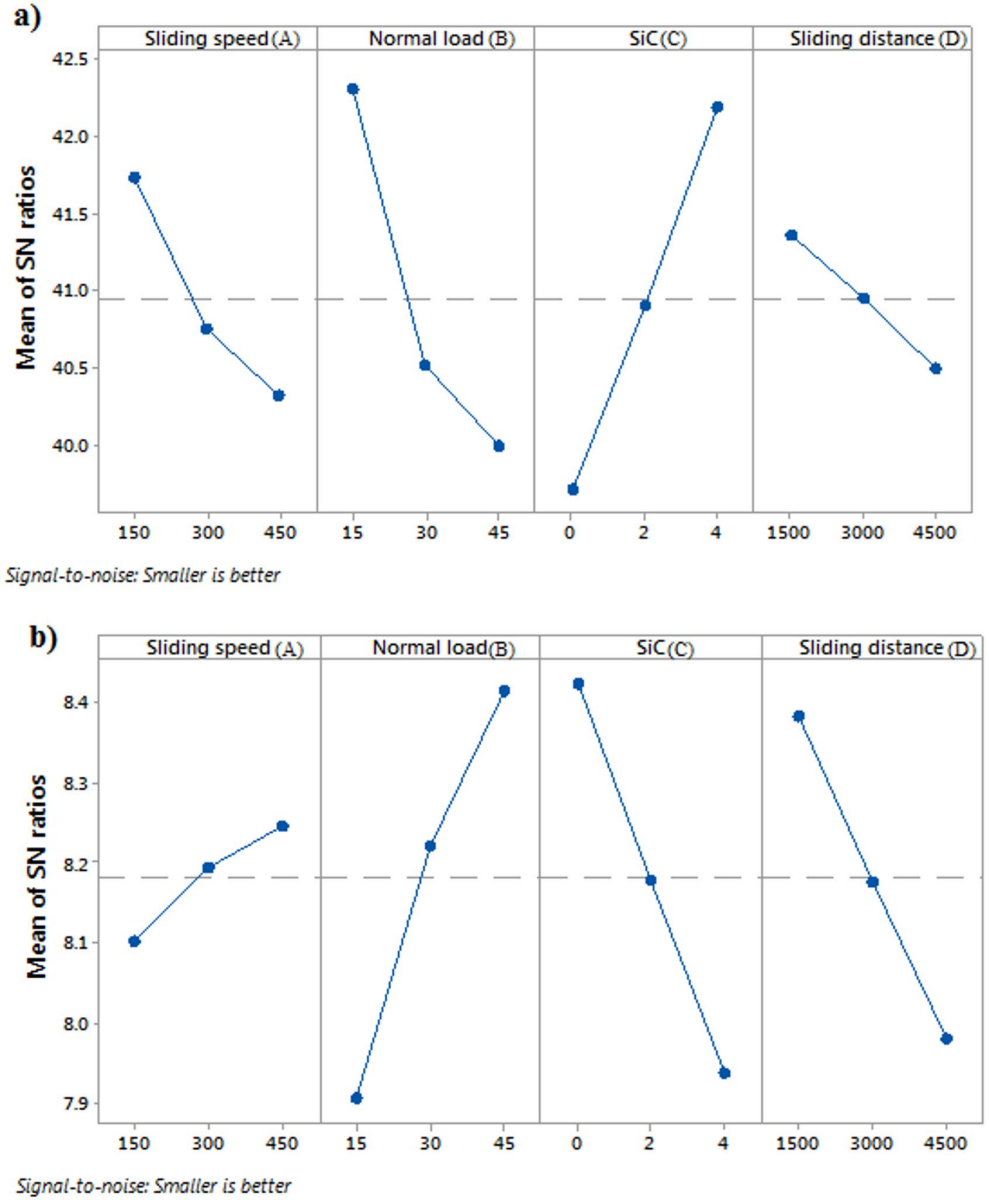
Main effect plot of S/N ratio (**a**) Wear rate (**b**) COF.

Figures 3 provide the main effect plot, offering insights into the wear rate and COF of SiC reinforced AlSi-1.5Mg composite. The main effects plot for SN ratios, where a higher SN ratio indicates a lower wear rate, provides clear insights into the impact of sliding speed, normal load, SiC content, and sliding distance on wear behavior under the “smaller is better” condition. Figure 3a shows that as sliding speed increases from 150 to 450, the SN ratio decreases, meaning that higher sliding speeds result in increased wear, with the lowest wear rate occurring at the slowest speed (150 RPM). Similarly, the normal load has a significant effect, with the highest SN ratio observed at the lowest load (15 N), while increasing the load to 45 N reduces the SN ratio and increases wear. SiC reinforcement plays a crucial role in reducing wear, as the SN ratio is lowest at 0% SiC, indicating the highest wear rate, and increases as SiC content rises to 4%, resulting in improved wear resistance. Finally, sliding distance also affects wear, with longer distances (4500 m) leading to lower SN ratios and greater wear, while shorter distances (1500 m) produce higher SN ratios and lower wear. In summary, to minimize wear and achieve the best results, the optimal conditions are low sliding speeds, low normal loads, high SiC content, and shorter sliding distances, each of which contributes to higher SN ratios and reduced wear. Figure 3b shows that increasing the sliding speed from 150 to 350 RPM leads to a rise in the SN ratio, indicating a reduction in COF, which suggests improved friction performance at higher speeds. Similarly, increasing the normal load from 15 to 45 N results in a higher SN ratio, implying that the COF decreases as the load increases, enhancing the material’s frictional characteristics under heavier loads. The inclusion of SiC also has a positive impact, as higher SiC content (from 0 to 4 wt%) leads to an increase in the SN ratio, indicating a lower COF and thus better wear properties. However, as the sliding distance increases from 1500 to 4500 m, the SN ratio decreases, implying that the COF increases over longer distances, likely due to the effects of wear and surface degradation. Overall, the results suggest that higher sliding speeds, greater normal loads, and the inclusion of SiC improve frictional performance, while extended sliding distances may lead to higher friction and wear.

**Fig. 4:**
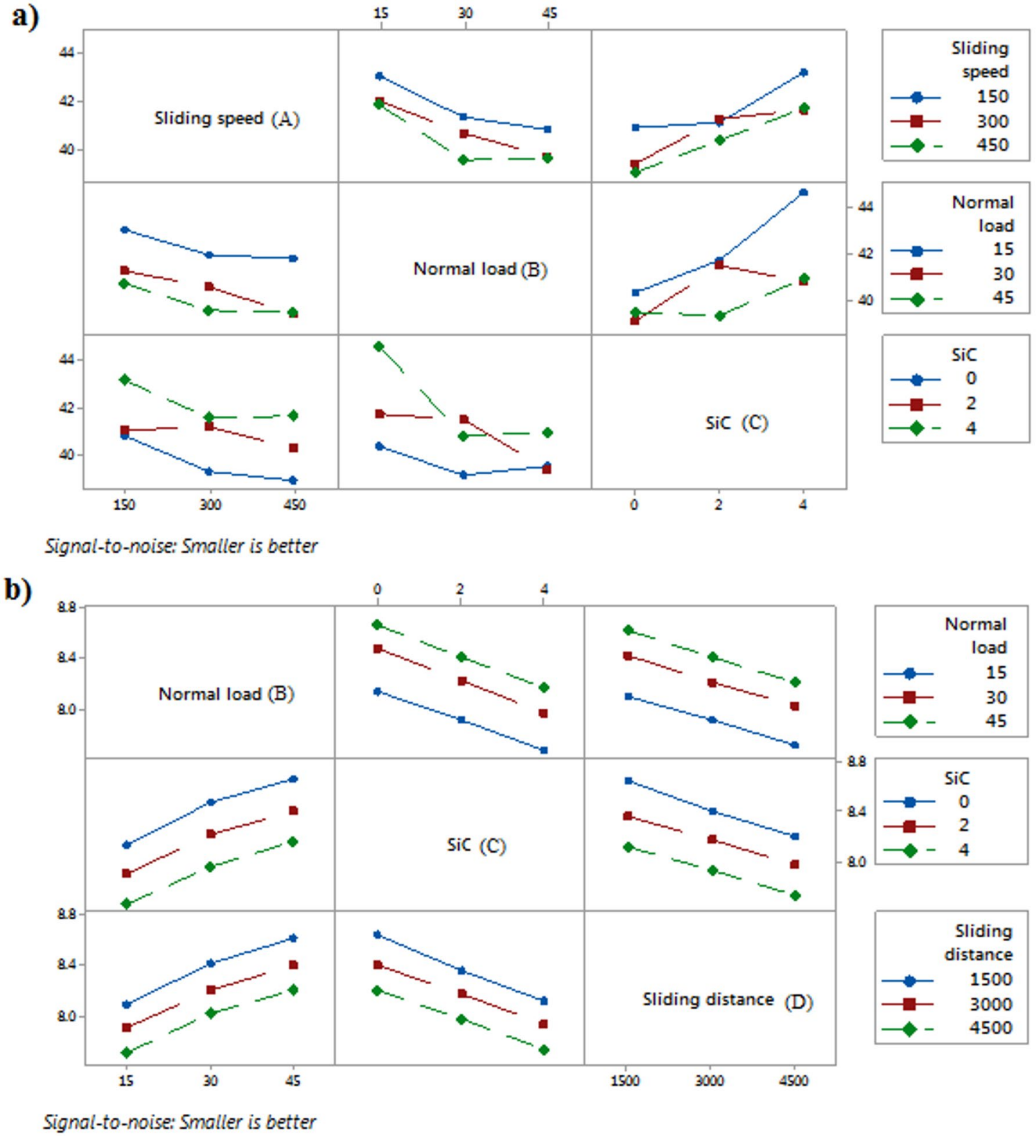
Interaction plot of S/N ratio (**a**) wear rate, (**b**) COF.

Figure 4 displays the interaction plot for SiC reinforced AlSi-1.5Mg composite in relation to the wear rate and COF. The interaction plot for SN ratios, where a higher SN ratio indicates a lower wear rate, reveals important insights about the effects of sliding speed, normal load, and SiC reinforcement on wear behavior. Figure 4a shows that at lower sliding speeds (150RPM), the SN ratios are generally higher, suggesting lower wear rates. As sliding speed increases, particularly at 450RPM, the SN ratio decreases, indicating higher wear rates. Similarly, the normal load has a significant impact on wear, with lower loads (15 N) producing higher SN ratios, which corresponds to reduced wear. As the normal load increases to 45 N, the SN ratios drop, implying that higher loads lead to more wear. The interaction between sliding speed and SiC content also highlights that higher sliding speeds, combined with increased SiC reinforcement (4 wt%), result in lower SN ratios, suggesting greater wear. Conversely, at lower sliding speeds and SiC contents, the wear rate is minimized, as indicated by higher SN ratios. Furthermore, the normal load and SiC content interaction shows that lower loads and minimal SiC reinforcement (0 wt%) result in better performance, reflected by higher SN ratios and lower wear rates. In summary, to achieve optimal wear resistance, the ideal conditions are low sliding speeds, low normal loads, and minimal SiC reinforcement, as these parameters lead to higher SN ratios and thus reduced wear rates. The Fig. 4b reveals how the combined effects of normal load, SiC content, and sliding distance influence the COF. Increasing the normal load from 15 to 45 N consistently leads to higher SN ratios, indicating a reduction in COF, with the impact being more significant when SiC content is higher. This suggests that the frictional performance improves under higher loads, especially when more SiC is present in the composite. However, as sliding distance increases from 1500 to 4500 m, SN ratios decrease across all normal loads, meaning the COF increases with longer sliding distances, and the effect of load on COF diminishes. Additionally, higher SiC content leads to lower COF at all sliding distances, but its effectiveness is most pronounced at shorter distances. Over extended sliding distances, the benefit of SiC in reducing COF lessens, likely due to increased wear. In summary, while higher normal loads and SiC content generally improve frictional performance, longer sliding distances tend to counteract these benefits by increasing COF due to wear over time.

SiC reinforcement generally improves wear resistance due to its hardness and load-bearing capacity, its influence on the COF is more complex^57,58^. The increase in SiC content raises the surface roughness and asperity interaction during sliding, which can lead to higher resistance to motion and, consequently, a higher COF. At the same time, the presence of hard SiC particles effectively protects the softer aluminum matrix from severe material removal, thereby reducing the wear rate^55,59^. Thus, the wear rate decreases with increasing SiC reinforcement, whereas the COF may not follow the same trend and can increase due to enhanced surface interactions^39,60,61^.

###  ANOVA

Table 5 presents the results of the ANOVA of wear rate for SiC reinforced AlSi-1.5Mg composite. The ANOVA results for the SN ratio related to the wear rate provide important insights into the impact of different sliding parameters and their interactions on the wear performance^28,62^; Mohammed, et al., 2024). SiC content is the most significant factor, contributing 32.04% to the variation in wear rate, demonstrating that incorporating hard SiC particles significantly enhances wear resistance by improving the composite’s hardness and reducing material loss. Normal load is also a major factor, contributing 30.98%, indicating that higher loads lead to increased wear, but this effect is mitigated by SiC reinforcement. Sliding speed plays a notable role, accounting for 10.99% of the variation, as higher speeds typically result in more frictional heat and greater wear, though this can be controlled by optimizing SiC content. Sliding distance has a smaller impact, contributing 3.91%, showing that although longer sliding distances increase wear, their influence is less pronounced compared to other factors. Significant interactions between factors are also observed. The interaction between normal load and SiC (17.28%) shows that the wear-reducing effect of SiC is more pronounced under higher loads. The interaction between sliding speed and SiC (3.40%) highlights that the presence of SiC becomes more effective in reducing wear at higher sliding speeds. The interaction between sliding speed and normal load has a minor influence, contributing 1.38%, indicating that their combined effect on wear rate is relatively small. The high F-values and P-values of 0.000 for all factors and interactions confirm their statistical significance, and the minimal error (0.03%) indicates that the experimental setup was reliable, with the variation in wear rate being primarily attributed to the control factors. In conclusion, SiC content and normal load are the most critical factors affecting wear rate, with higher SiC content and optimized load conditions significantly reducing wear. Sliding speed and sliding distance also influence wear, but to a lesser extent. Optimizing these parameters, particularly the SiC content and load, is crucial for enhancing wear resistance in Al-Si-1.5Mg MMCs, making them more suitable for high-wear industrial applications.

**Table 5:** ANOVA of S/N ratio (wear rate).

Source	DOF	Adj SS	Adj MS	F-Value	*P*-Value	*P*%
Sliding speed	2	9.3541	4.6771	1243.28	0.000	10.99
Normal load	2	26.3716	13.1858	3505.11	0.000	30.98
SiC	2	27.2792	13.6396	3625.74	0.000	32.04
Sliding distance	2	3.3265	1.6633	442.13	0.000	3.91
Sliding speed*Normal load	4	1.1707	0.2927	77.80	0.000	1.38
Sliding speed*SiC	4	2.8958	0.7239	192.44	0.000	3.40
Normal load*SiC	4	14.7092	3.6773	977.52	0.000	17.28
Error	6	0.0226	0.0038			0.03
Total	26	85.1296				100.00

R-sq = 99.97, R-sq(adj) = 99.89%

Level of significance (α) = 0.05 (5%) or Confidence level = 0.95 (95%)

Table 6 presents the results of the ANOVA of COF conducted for the SiC reinforced AlSi-1.5Mg composite. The ANOVA results for the SN ratio related to the COF reveal the significant influence of sliding parameters on the frictional behavior of SiC-reinforced Al-Si eutectic MMCs. The most critical factor affecting COF is the normal load, contributing 38.51% to the total variation. This suggests that as the normal load increases, the COF rises significantly due to greater surface contact and friction between the composite and the sliding surface. SiC content also plays a crucial role, accounting for 34.46% of the variation, where the inclusion of SiC particles enhances the hardness of the composite, reducing material deformation under load and, consequently, the COF. Sliding distance is another important factor, contributing 23.51% to the variation. Longer sliding distances increase the wear and friction exposure, resulting in a higher COF, though its impact is less pronounced than normal load and SiC content. Among the interaction effects, the interaction between SiC content and sliding distance is statistically significant, contributing 0.19%, indicating that SiC’s effect on reducing COF becomes more prominent over extended sliding distances. However, other interaction terms, such as normal load × SiC and normal load × sliding distance, have negligible contributions and are not statistically significant, suggesting that these interactions do not significantly impact COF. The error in the model is minimal, contributing only 0.06% to the total variation, underscoring the accuracy of the results. With an R-squared value of 99.94% and an adjusted R-squared of 99.73%, the model explains nearly all the variation in COF. In conclusion, normal load, SiC content, and sliding distance are the key factors influencing COF, and optimizing these parameters, especially by increasing SiC content and carefully controlling the normal load, can effectively reduce friction and enhance the wear performance of SiC-reinforced Al-Si-1.5Mg MMCs in dry sliding conditions.

**Table 6:** ANOVA of S/N ratio (COF).

Source	DOF	Adj SS	Adj MS	F-Value	*P*-Value	*P* %
Sliding speed	2	0.09694	0.048472	152.22	0.000	3.15
Normal load	2	1.18351	0.591754	1858.35	0.000	38.51
SiC	2	1.05907	0.529536	1662.96	0.000	34.46
Sliding distance	2	0.72254	0.361272	1134.54	0.000	23.51
Normal load*SiC	4	0.00210	0.000537	1.69	0.270	0.07
Normal load*Sliding distance	4	0.00120	0.000301	0.95	0.499	0.04
SiC*Sliding distance	4	0.00593	0.001481	4.65	0.047	0.19
Error	6	0.00191	0.000318			0.06
Total	26	3.07326				100.00

R-sq = 99.94%, R-sq(adj) = 99.73%

Level of significance (α) = 0.05 (5%) or Confidence level = 0.95 (95%)

**Fig. 5:**
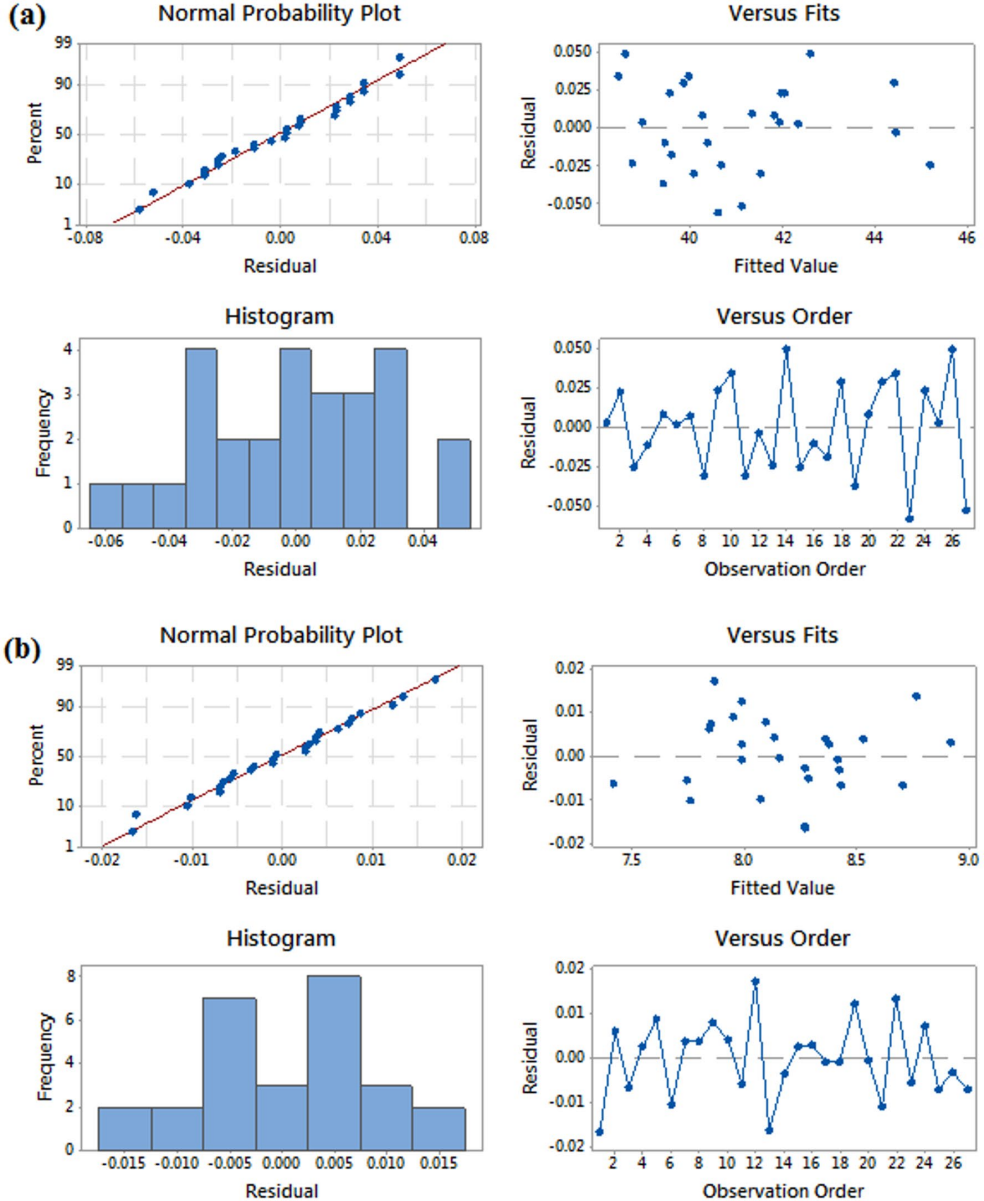
Residual plot of S/N ratio (**a**) wear rate, (**b**) COF.

Figure 5a, b presents the residual plots for the SN ratios of wear rate and COF, it provide a comprehensive analysis of the model fit related to wear rate. The normal probability plot reveals that the points closely follow a straight line, indicating that the residuals are approximately normally distributed, thereby satisfying the model’s assumptions of normality and suggesting a good fit for predicting wear rate^1^. Additionally, the residuals versus fitted values plot shows no clear pattern, with residuals scattered randomly around zero, which indicates that the model adequately explains the variability in the wear rate without signs of non-linearity or unequal error variance (heteroscedasticity). The histogram of residuals further reinforces this notion, appearing somewhat symmetric and free of extreme outliers, which supports the model’s reliability in predicting wear rate. Moreover, the plot of residuals versus observation order exhibits no clear trends or cyclical patterns, signifying that the residuals are independent over time, confirming the model’s consistency. Overall, the analysis of the residual plots suggests that the model used to evaluate the SN ratios for wear rate is appropriate, with normally distributed and independent residuals, and no major issues regarding model fit detected. This indicates that the factors considered in the analysis effectively explain the variability in the wear rate.

### Taguchi confirmation

To validate the effectiveness of the optimal parameters identified through the Taguchi method, a confirmation test was conducted. This step is essential to ensure that the selected levels of control variables produce results consistent with the model’s predictions, thereby confirming the accuracy and practical relevance of the optimization process^45,48^. The prediction of wear performance, specifically the wear rate and COF were carried out using a S/N ratio-based model developed from experimental data. This approach enables estimation of expected responses without requiring extensive additional testing^64,46,33^. The predictive equation for optimum parameter A_1_, B_1_, C_3_ and D_1_ for wear rate was used to calculate the expected S/N ratio, which was then compared with experimental results to assess the validity of the optimization. The equation is presented in Eq. (1).

1 $$\begin{aligned} \bar{\eta }_{{opt}} & = \bar{T} + \left( {\bar{A}_{1} - \bar{T}} \right) + \left( {\bar{B}_{1} - \bar{T}} \right) + \left( {\bar{C}_{3} - \bar{T}} \right) \\ & + \left( {\bar{D}_{1} - \bar{T}} \right) + \left[ {\left( {\bar{A}_{1} \bar{B}_{1} - \bar{T}} \right) - \left( {\bar{A}_{1} - \bar{T}} \right) - \left( {\bar{B}_{1} - \bar{T}} \right)} \right] \\ & + \left[ {\left( {\bar{A}_{1} \bar{C}_{3} - \bar{T}} \right) - \left( {\bar{A}_{1} - \bar{T}} \right) - \left( {\bar{C}_{3} - \bar{T}} \right)} \right] \\ & + \left[ {\left( {\bar{B}_{1} \bar{C}_{3} - \bar{T}} \right) - \left( {\bar{B}_{1} - \bar{T}} \right) - \left( {\bar{C}_{3} - \bar{T}} \right)} \right]~ \\ \end{aligned}$$

Where, $\:{\stackrel{-}{\text{A}}}_{1},\:{\stackrel{-}{\text{B}}}_{1},\:{\stackrel{-}{\text{C}}}_{3}\:\text{a}\text{n}\text{d}\:{\stackrel{-}{\text{D}}}_{1}$- mean response, $\:\stackrel{-}{\text{T}}$= overall average of experimental S/N ratio. Neglecting the interactions of the least significant factor, sliding distance (D), Eq. (1) can be further reduced to Eq. (2).

2 $$\:{\stackrel{-}{{\upeta\:}}}_{\text{o}\text{p}\text{t}}={\stackrel{-}{\text{A}}}_{1}-{\stackrel{-}{\text{B}}}_{1}-{\stackrel{-}{\text{C}}}_{3}-{\stackrel{-}{\text{D}}}_{1}+{\stackrel{-}{\text{A}}}_{1}{\stackrel{-}{\text{B}}}_{1}+{\stackrel{-}{\text{A}}}_{1}{\stackrel{-}{\text{C}}}_{3}+{\stackrel{-}{\text{B}}}_{1}{\stackrel{-}{\text{C}}}_{3}$$

For the optimum parameter A_3_, B_3_, C_1_ and D_1_ for COF, the predictive equation can be written as Eq. (3).

3 $$\begin{aligned} \bar{\eta }_{{opt}} & = \bar{T} + \left( {\bar{A}_{3} - \bar{T}} \right) + \left( {\bar{B}_{3} - \bar{T}} \right) + \left( {\bar{C}_{1} - \bar{T}} \right) + \left( {\bar{D}_{1} - \bar{T}} \right) \\ & + \left[ {\left( {\bar{B}_{3} \bar{C}_{1} - \bar{T}} \right) - \left( {\bar{B}_{3} - \bar{T}} \right) - \left( {\bar{C}_{1} - \bar{T}} \right)} \right] \\ & + \left[ {\left( {\bar{B}_{3} \bar{D}_{1} - \bar{T}} \right) - \left( {\bar{B}_{3} - \bar{T}} \right) - \left( {\bar{D}_{1} - \bar{T}} \right)} \right] \\ & + \left[ {\left( {\bar{C}_{1} \bar{D}_{1} - \bar{T}} \right) - \left( {\bar{C}_{1} - \bar{T}} \right) - \left( {\bar{D}_{1} - \bar{T}} \right)} \right] \\ \end{aligned}$$

Where, $\:{\stackrel{-}{\text{A}}}_{3},\:{\stackrel{-}{\text{B}}}_{3},\:{\stackrel{-}{\text{C}}}_{1}\:\text{a}\text{n}\text{d}\:{\stackrel{-}{\text{D}}}_{1}$- mean response for control factors and interactions at designated levels, $\:\stackrel{-}{\text{T}}$= overall average of experimental S/N ratio. Ignoring the interactions of the least significant factor sliding velocity (A), Eq. (3) can be further simplified to Eq. (4).

4 $$\:{\stackrel{-}{{\upeta\:}}}_{\text{o}\text{p}\text{t}}={\stackrel{-}{\text{A}}}_{3}-{\stackrel{-}{\text{B}}}_{3}-{\stackrel{-}{\text{C}}}_{1}-{\stackrel{-}{\text{D}}}_{1}+{\stackrel{-}{\text{B}}}_{3}{\stackrel{-}{\text{C}}}_{1}+{\stackrel{-}{\text{B}}}_{3}{\stackrel{-}{\text{D}}}_{1}+{\stackrel{-}{\text{C}}}_{1}{\stackrel{-}{\text{D}}}_{1}$$

Table 7 presents a comparison between the experimental results and the values predicted by the Taguchi-based model. The observed S/N ratios, wear rate, and COF closely matched the predicted values, indicating strong agreement between the model and actual performance outcomes. The confirmation test revealed a maximum deviation of only 2.01% for both wear rate and COF, demonstrating the reliability and accuracy of the optimization process.

**Table 7:** Taguchi confirmation.

Level		Predict	Experimental	Error (%)
		A_1_B_1_C_3_D_1_	A_1_B_1_C_3_D_1_	
Wear rate	S/N ratio(dB)	46.0791	47.0231	2.01
	Wear rate (mm^3^/Nm)	0.004569	0.004598	0.63

Level		Predict	Experimental	Error (%)
		A_3_B_3_C_1_D_1_	A_3_B_3_C_1_D_1_	
COF	S/N ratio(dB)	8.92708	8.99834	0.79
	COF	0.357356	0.362451	1.41

### Worn surfaces characterization

**Fig. 6:**
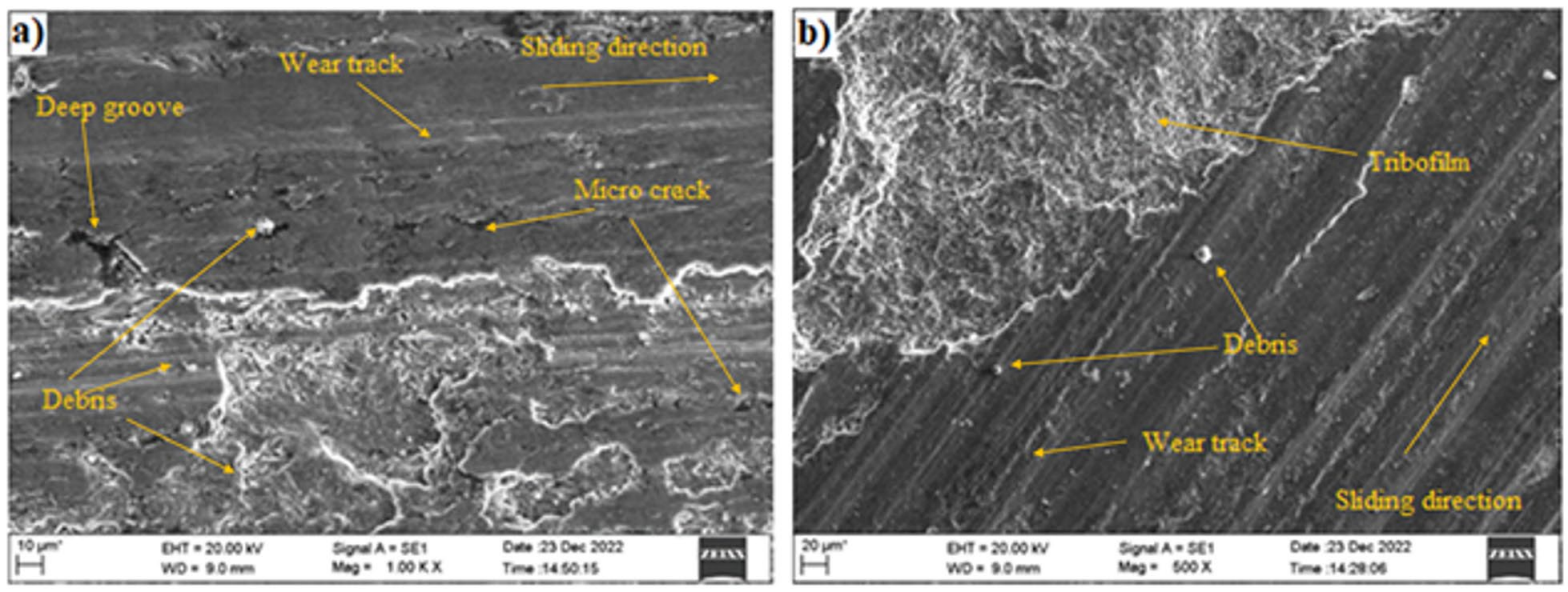
SEM images showing worn surfaces of Al–Si–Mg composites under sliding conditions of 450 RPM, 45 N load, and 4500 m sliding distance: (**a**) unreinforced (0% SiC), (**b**) reinforced with 4% SiC.

The comparison of the SEM micrographs in Fig. 6a (speed 450 RPM, load 45 N, sliding distance 4500 m, 0% SiC) and Fig. 6b (same conditions at 4% SiC) highlights the significant influence of SiC reinforcement on the dry sliding wear behavior of Al–Si–Mg metal matrix composites. The worn surface of the unreinforced composite in Fig. 6(a) exhibits extensive surface damage, including deep grooves, delamination, severe plastic deformation, and large wear debris, indicating a dominant adhesive–abrasive wear mechanism due to the low hardness and limited load-bearing capacity of the aluminum matrix ^59,65^. Localized softening and smearing caused by frictional heating further contribute to increased material loss ^66,67^. In contrast, Fig. 6b, representing the SiC-reinforced composite, shows a smoother and more uniform surface with finer grooves, reduced delamination, and minimal material pull-out, demonstrating the beneficial effect of SiC particles in enhancing hardness and stiffness ^68,69^. Fractured and embedded SiC particles on the worn surface act as a protective tribofilm, lowering friction and controlling wear progression ^55,60,68^. This comparison confirms that even a small addition of SiC significantly improves the tribological performance, consistent with the observed reductions in wear rate and coefficient of friction.

## Conclusion

This study examined the influence of sliding parameters on the dry sliding wear behavior of SiC-reinforced Al–Si eutectic MMCs containing traces of Mg. The results show that sliding speed, normal load, sliding distance, and SiC content have a significant impact on wear performance. Increasing the SiC content enhanced hardness and improved wear resistance by reducing friction and surface damage. The presence of trace amounts of magnesium contributed to stronger interfacial bonding between the SiC particles and the matrix, improving wettability and stability under load.

ANOVA results indicated that the wear rate was primarily governed by SiC content (32.04%), normal load (30.98%), and sliding speed (10.99%), while the coefficient of friction (COF) was mainly affected by normal load (38.51%), SiC content (34.46%), and sliding distance (23.51%). The Taguchi method further emphasized the importance of parameter optimization in minimizing wear under high-stress conditions. These findings highlight the potential of the developed composites for use in sectors such as automotive, aerospace, and marine industries, where components are routinely subjected to severe wear environments.

The present work was limited to dry sliding wear tests at room temperature. Conditions such as elevated temperatures, lubricated wear, and extended statistical analyses were not considered. Future studies addressing these aspects would provide a more comprehensive understanding of the wear mechanisms and broaden the scope for applying these composites in demanding service environments.

## Data Availability

Corresponding author agrees to provide the data upon reasonable request.
